# MUSCLE SELECTION AND DOSING IN PATIENTS UNDERGOING TREATMENT WITH ABOBOTULINUMTOXINA FOR LOWER LIMB SPASTICITY IN REAL-WORLD PRACTICE

**DOI:** 10.2340/jrm.v57.42605

**Published:** 2025-02-07

**Authors:** Richard D. ZOROWITZ, Jorge JACINTO, Stephen ASHFORD, Mathieu BENETEAU, Pascal MAISONOBE, Christian HANNES, Alberto ESQUENAZI

**Affiliations:** 1 MedStar Health, Washington and Georgetown University School of Medicine, Washington, DC, USA; 2 Centro de Medicina de Reabilitação de Alcoitão, Serviço de Reabilitação de Adultos 3, Estoril, Portugal; 3 London North West University Healthcare NHS Trust, Regional Hyper-Acute Rehabilitation Unit, Northwick Park Hospital, London, UK; 4 Ipsen, Boulogne-Billancourt, France; 5 Ipsen, Munich, Germany; 6 MossRehab Jefferson Health, Elkins Park, PA, USA

**Keywords:** abobotulinumtoxinA, botulinum toxin A, muscle dosing, rehabilitation

## Abstract

**Objective:**

Describe abobotulinumtoxinA (aboBoNT-A) dosing parameters in the real-world management of lower limb spasticity (LLS).

**Methods:**

Prospective, observational study (NCT04050527) following ambulatory adults with unilateral LLS treated with aboBoNT-A.

**Results:**

The effectiveness population included 384 adults with LLS. Across the study, total lower limb doses were higher in patients who received only lower limb injections (*n* = 131, median 771U) than those who also received ≥ 1 upper limb injection (*n* = 253, 567U). Total doses increased over subsequent cycles in both subgroups. Six muscles (gastrocnemius medial and lateral heads, soleus muscle, tibialis posterior, flexor digitorum longus, and flexor hallucis longus) were identified as the main targets for the treatment of LLS; other lower limb muscles were injected in fewer than 15% of patients. The most frequent therapy interventions (mean ± SD of 1.8 ± 1.3h/week with a qualified therapist and 5.3 ± 5.9h/week self-rehabilitation in Cycle 1) were task-specific practice, passive stretch, strength training, and positioning.

**Conclusions:**

This study demonstrates how a diversity of muscle patterns are currently treated in routine practice where the primary goal was related to the lower limb and highlights important issues for further debate, such as potential underdosing and the need to balance upper and lower limb priorities when devising a treatment plan.

## LAY ABSTRACT

This paper describes information on how clinicians working in specialist neurorehabilitation centres routinely treat their patients presenting with lower limb spasticity (patients with thigh, leg, and/or foot muscles that are stiff, tight, and difficult to control due to a neurological injury such as stroke) with the botulinum toxin known as abobotulinumtoxinA. The study found that the doses of abobotulinumtoxinA used to treat patients able to walk at least 5 steps often are lower than used in prior clinical trials. Six muscles were identified as being most frequently injected for lower limb spasticity, with other muscles being much less frequently targeted. By comparing injection practices in different subgroups of patients, the authors observed that a possible reason for the lower-than-expected dosing could be the frequent need to manage upper limb (shoulder, upper arm, forearm, wrist, and hand) spasticity at the same time. As such, the results of this study highlight potential areas for education and further study to maximize the therapeutic benefits of this treatment.

It is estimated that approximately one-third of stroke survivors develop lower limb spasticity (LLS) affecting the hip, knee, and/or ankle (1). Depending on the underlying aetiology and location of the brain lesion, stroke survivors may develop a variety of lower limb spasticity patterns. The most commonly involved muscles are the ankle plantar flexors, followed by hip adductors, knee extensors, knee flexors, and hip internal rotators (1). The most common pattern of post-stroke walking limitation is gait characterized by asymmetry associated with ankle plantar flexion and inversion (2). While spastic equinovarus deformities with toe curling predominate, equinus, varus, and striatal toe deformities are also common (3).

The cornerstone of functional spasticity management is physical rehabilitation, often facilitated by pharmacological intervention. Guidelines recommend botulinum toxin-A (BoNT-A) in the management of LLS as part of routine practice (4, 5). The American Academy of Neurology currently recommends abobotulinumtoxinA (aboBoNT-A) and onabotulinumtoxinA due to their established efficacy and safety, with other formulations having insufficient evidence (4). Pivotal studies showed that repeated administrations of aboBoNT-A over 1 year are well tolerated, improve walking speed, and increase the likelihood of achieving community ambulation (6).

We have previously reported the primary effectiveness results of this large observational study following longitudinal goal attainment in ambulatory adult patients after ≥ 1 aboBoNT-A injection for LLS in a routine clinical setting (7). Patients generally achieved their primary goals (mean cumulated GAS-leg T score of 48.2 [47.4, 48.9]), and patients injected using an instrumented guidance technique (i.e., electromyography, electrostimulation, or ultrasound) in addition to anatomical landmarks at baseline were more likely to attain their primary treatment goals during Cycle 1 than those injected without such guidance (odds ratio: 1.9 [95% CI 1.1, 3.1], *p* = 0.02) (7). Because appropriate muscle selection and dosing are prerequisites for treatment success, clinicians must have an understanding of how to tailor dosing within a safe and efficacious range. Aside from basic dosing recommendations given in the prescribing information, there currently is only limited information available to guide decision-making and, consequently, the use of BoNT-A for LLS often is influenced by other factors, such as access to treatment and injector training (8). To fill this gap, we present a detailed description of injection parameters used within this real-world “routine-practice” study conducted at centres across the United States (US), Australia, Brazil, Canada, Russia, and Europe.

## METHODS

### Study design and participants

Full details of the study methods have been published previously (7). In brief, the AboLiSh study was a prospective, longitudinal (16-month), observational cohort study (NCT04050527) conducted at 46 expert neurorehabilitation centres. The study was conducted in compliance with the International Society for Pharmacoepidemiology (ISPE) Guidelines for Good Pharmacoepidemiology Practices (GPP). Independent Ethics Committee/ Institutional Review Board approval was obtained prior to each centre initiation, and written informed consent was obtained prior to patient enrolment.

Adult patients (≥ 18 years) with unilateral LLS able to take ≥ 5 steps with or without assistance were treated with aboBoNT-A in accordance with local prescribing guidelines to achieve individualized treatment goals. The decision to prescribe aboBoNT-A (Dysport, Ipsen, Wrexham, UK) was made prior to, and independently of, enrolment in the study, and investigators were free to tailor injection parameters (dosing, muscle patterns, treatment intervals etc.) to patients’ individual needs. There was no mandated schedule of assessment. Patients with severe limitations in passive range of motion/contractures in the affected limb, limb surgery, or intrathecal baclofen therapy within the prior 3 months, a progressive neurological condition, or who had received BoNT-A within 12 weeks prior to study enrolment were excluded.

All patients underwent a comprehensive clinical spasticity assessment at baseline/first injection visit, and the full details of the injections (i.e., muscles selected, injected dose, injected volume, number of injection sites, use of injection guidance technique) were collected in an electronic case report form. Data regarding non-pharmacological treatment were captured by the Lower Extremity Therapy Recording Schedule (LegTS) (9).

### Statistical analysis

The statistical analyses are descriptive and are presented for patients who received ≥ 1 BoNT-A injection and had ≥ 1 post-baseline Goal Attainment Scaling (GAS) assessment (effectiveness population). Predefined subgroups included patients who received lower limb injections only and those who received lower limb and ≥ 1 upper limb injections. Mean and standard deviation (mean ± SD) or median measures were used to summarize continuous variables, and absolute and relative frequencies expressed as percentages (%) are presented for categorical information.

## RESULTS

Baseline demographics and clinical characteristics of the 384 patients included in the effectiveness population have been described previously (7) and are indicated in Table SI. Most patients were male (66.4%), with a mean ± SD age of 53.9 ± 13.8 years, and the majority (97.4%) had cerebral infarcts or haemorrhages. Overall, 85.4% of patients were also reported as having upper limb spasticity (ULS). Just over three-quarters of patients (*n* = 288, 76.0%) had a history of previous injections with BoNT-A, with 65% having been treated previously for LLS. The median duration of BoNT-A treatment prior to study enrolment was 3.0 years, with the longest treatment period being 22.9 years.

### AbobotulinumtoxinA injection parameters

The median (range) injection interval across all treatment cycles was 17.0 weeks (12.1–69.3). The median total injected dose of aboBoNT-A in the lower limb was 600 U (range 100–1,475 U), which was injected into a median of 4 muscles (range 1–8). Guidance techniques were used in 77% of Cycle 1 injections (Table I), with some patients being injected using ≥ 1 method simultaneously. While there were differences in reconstitution routines, the modal aboBoNT-A dilution was 200 U/mL.

**Table I:** AbobotulinumtoxinA treatment for lower limb spasticity (effectiveness population)

Parameter	*n* = 384
Total dose for lower limb (U) Mean ± SD Median (Q1, Q3)	665 ± 278 600 (478, 900)
Number of injected lower limb muscles Mean ± SD Median [Q1, Q3]	4.0 ± 1.4 4 (3, 5)
Number of injection points (lower limbs) Mean ± SD Median [Q1, Q3]	6.7 ± 3.1 6.0 (4.7, 8.7)
Duration of injection cycles (weeks) Mean ± SD Median [Q1, Q3]	18.3 ± 6.1 17.04 (14.7, 18.4)
Annualised number of administrations for LLS Mean ± SD Median [Q1, Q3]	2.9 ± 0.8 3.0 (2.8, 3.5)
Use of injection guidance for lower limbs during Cycle 1, *n* (%) EMG Electrical stimulation Ultrasound None	126 (42.6%) 94 (31.8%) 126 (42.6%) 88 (22.9%)

**Fig. 1:**
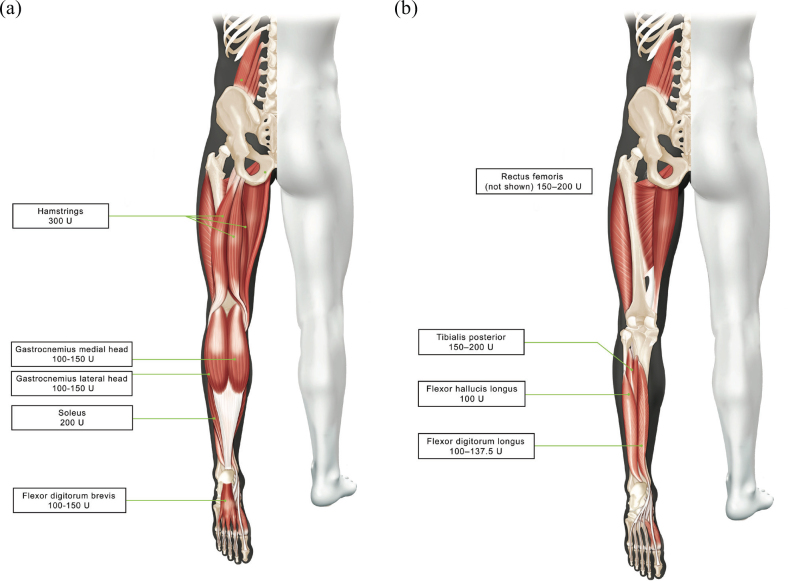
Median doses per muscle used in Cycles 1–5 (muscles injected in >10% of patients) (A) superficial muscles; (B) deep muscles.

**Fig. 2:**
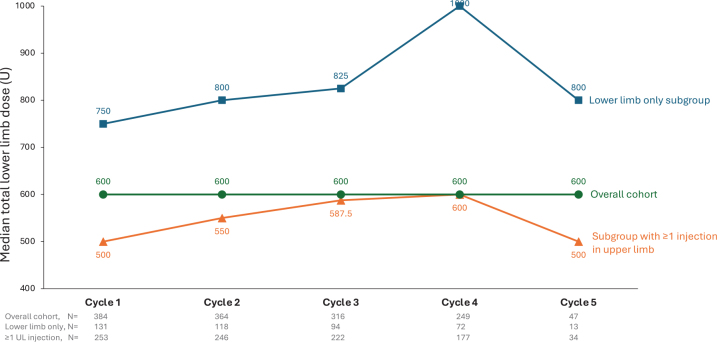
Total lower limb doses by cycle (per subgroup and overall).

Fig. 1 lists the range of median aboBoNT-A doses used per muscle across 5 cycles. The most commonly injected muscles (injected in ≥ 20% of the population) were the gastrocnemius medial and lateral heads, soleus, tibialis posterior, flexor digitorum longus, and flexor hallucis longus). Across the study, total doses for the lower limb were higher in patients who received only lower limb injections (*n* = 131, median [Q1, Q3] dose of 771 [500, 1,000] U) than those who also received ≥ 1 upper limb injection (*n* = 253, 567 [450, 783] U). However, both subgroups had a median of 4 muscles injected per cycle. Total lower limb doses tended to increase over subsequent cycles in both subgroups (Fig. 2). However, this incremental increase was not seen when the whole cohort was analysed.

Table II provides Cycle 1 dosing parameters for patients injected in the lower limb only versus those who also received ≥ 1 injection for ULS. In Cycle 1, dosing between the 2 subgroups generally was similar for most muscles except for the flexor digitorum longus, hamstrings, and rectus femoris, which were injected at higher doses in patients who received only lower limb injections. Other muscles that also were injected at higher doses (lower limb only subgroup vs those with ≥ 1 upper limb injection) included the: extensor hallucis longus (125 U [6.1%] vs 75 U [3.2%]), adductor longus (200 U [3.8%] vs 150 U [0.4%]), flexor hallucis brevis (100 U [2.3%] vs 50 U [3.6%]), quadriceps (145 U [1.5%] vs 100 U [3.2%]), and anterior tibialis (300 U [3.1%] vs 100 U [2.0%]). Results were similar for Cycles 2–5. When both upper and lower limbs were injected in Cycle 1 (*n* = 236), the median (range) total body dose of aboBoNT-A was 1400 U (1,000–1,500 U).

**Table II:** AbobotulinumtoxinA Cycle 1 dosing parameters overall and for the muscles injected in ≥ 10% of the overall population

Cycle 1 parameter	Injected in lower limb only (*n* = 131)	Received ≥ 1 injection in upper limb (*n* = 253)
Total dose for lower limb (U) Mean ± SD Median (Q1, Q3)	727 ± 297 750 (500, 1,000)	607 ± 266 500 (400, 800)
Total body dose (U) Mean ± SD Median (Q1, Q3)	727 ± 297 750 (500, 1,000)	1,243 ± 356 1400 (1,000, 1,500)
Gastrocnemius medial head Median (Q1, Q3)	*n* = 111, 150 (100, 200)	*n* = 210 150 (100, 200)
Gastrocnemius lateral head Median (Q1, Q3)	*n* = 100 150 (100, 150)	*n* = 184 150 (100, 162.5)
Soleus Median (Q1, Q3)	*n* = 87 200 (150, 300)	*n* = 170 200 (150, 300)
Tibialis posterior Median (Q1, Q3)	*n* = 65 200 (150, 200)	*n* = 126 200 (100, 200)
Flexor digitorum longus Median (Q1, Q3)	*n* = 60 150 (100, 200)	*n* = 96 100 (100, 150)
Flexor hallucis longus Median (Q1, Q3)	*n* = 25 100 (100, 150)	*n* = 59 100 (100, 150)
Rectus femoris Median (Q1, Q3)	*n* = 12 200 (110, 300)	*n* = 41 180 (100, 300)
Hamstrings Median (Q1, Q3)	*n* = 26 375 (300, 400)	*n* = 25 200 (100, 300)
Flexor digitorum brevis Median (Q1, Q3)	*n* = 18 100 (100, 150)	*n* = 31 100 (50, 200)

Approximately one-quarter of patients were receiving concomitant spasticity and/or spasticity-related pain medications at study entry (Table III). Data regarding non-pharmacological treatment were captured by the LegTS, although, due to the observational nature of the study, there were considerable missing data. Table IV provides a breakdown of the therapy interventions per cycle. Most patients were treated in individual sessions with a qualified therapist or therapy assistant rather than in group sessions. The mean ± SD total amount of time that patients individually spent in therapy with a qualified therapist was 1.8 ± 1.3 hours per week in Cycle 1 but tended to increase to 2.2 ± 3.6 hours per week in Cycle 5 and may represent their severity. The mean ± SD total amount of time spent on self-practice (i.e., self-rehabilitation exercises without a therapist) was 5.3 ± 5.9 hours in Cycle 1 and 5.2 ± 3.1 hours in Cycle 5.

**Table III:** Concomitant pharmacological therapy at study entry

Parameter	*n* = 384
Concomitant medications for spasticity or related pain	98 (25.5%)
Systemic anti-spasticity medication	82 (21.4%)
Pain medications: neuropathic pain	35 (9.1%)
Pain medications: NSAIDs/paracetamol	27 (7.0%)
Opioids	7 (1.8%)
Phenol, alcohol or other neurolytic agents	3 (0.8%)

**Table IV:** Summary of therapy interventions per cycle

Intervention	Cycle 1 *n/N* (%)	Cycle 2 *n/N* (%)	Cycle 3 *n/N* (%)	Cycle 4 *n/N* (%)	Cycle 5 *n/N* (%)
Splinting	53/194 (27.3%)	51/202 (25.2%)	50/187 (26.7%)	43/165 (26.1%)	39/146 (26.7%)
Orthotic	70/194 (36.1%)	72/202 (35.6%)	60/187 (32.1%)	53/165 (32.1%)	48/146 (32.9%)
Serial casting	44/194 (22.7%)	40/202 (19.8%)	42/187 (22.5%)	33/165 (20.0%)	32/146 (21.9%)
Positioning of leg	134/194 (69.1%)	126/202 (62.4%)	107/187 (57.2%)	88/165 (53.3%)	71/146 (48.6%)
Passive stretch	148/194 (76.3%)	129/202 (63.9%)	114/187 (61.0%)	101/165 (61.2%)	84/146 (57.5%)
Electricalstimulation	45/194 (23.2%)	38/202 (18.8%)	29/187 (15.5%)	21/165 (12.7%)	25/146 (17.1%)
Strength training	128/194 (66.0%)	125/202 (61.9%)	108/187 (57.8%)	103/165 (62.4%)	88/146 (60.3%)
Task practice	146/194 (75.3%)	169/202 (83.7%)	159/187 (85.0%)	136/165 (82.4%)	122/146 (83.6%)
Missing	190	170	149	118	66

## DISCUSSION

In this study, 6 muscles (gastrocnemius medial and lateral heads, soleus muscle, tibialis posterior, flexor digitorum longus, and flexor hallucis longus) were identified as the main targets for the treatment of LLS and were consistent with treatment of equinus and/or varus foot as the most common pattern of LLS. On average, total aboBoNT-A doses for the lower limb were lower than expected (less than half of the maximum approved dose for LLS). However, 3 in every 5 patients also required injections into the upper limbs, and total lower limb doses were, on average, 200 U lower in patients who also received injections for upper limb spasticity. As expected for an ambulatory population, the most frequent therapy interventions were task-specific practice, passive stretch, strength training, and positioning (therapeutic or stretching position).

The median total lower limb dose of 600 U is less than half of the 1,500 U dose found in a Phase 3 pivotal study to reduce muscle tone in the gastrocnemius–soleus complex and with which repeated dosing was associated with an increase in walking speed (6). In that dose-ranging study, the lower total dose of 1,000 U produced significant effects vs placebo on reducing muscle tone in the soleus (6). Thus, it is possible that the broader population of patients enrolled in this study did not require the higher doses needed in the pivotal trial population. While we consider the doses injected into the gastrocnemius (150 U into the lateral and medial heads) relatively low given the size of the muscle and grade of tone or spastic responses, they were consistent with the current labels. While the US label restricts dosing in the gastrocnemius to 150 U per head (vs a maximum of 450 U in Europe), there was no evidence that the US sites drove the lower dosing. With regard to the soleus, the median dose of 200 U is well below the 330–500 U in the US label and 300–500 U in most European countries recommended as safe and efficacious. As the soleus is vital for efficient push-off during walking, and therefore walking velocity, injectors also may have been cautious to avoid an unveiling of underlying weakness, especially given that patients generally were expected to participate in walking task practice (the highest ranked physical intervention). Also of note, despite the lower doses used, the median injection interval of 17 weeks observed in this study is longer than the established duration of 12 weeks for BoNT-A and is more in line with an ULS observational study where the mean injection interval for patients treated with aboBoNT-A was 189.5 days, approximately 30 days longer than other BoNT-A products (*p*<0.001) (10). Because the duration of action is correlated with dose (11), our data indicate a yet unexplored potential for optimizing treatment outcomes and for increasing treatment intervals even further if optimal dosing (tailored to goals) is used. This would improve our ability to meet patients’ desires for less frequent injections (12) as well as to optimize management of clinical schedules and access to more patients.

The lower aboBoNT-A doses used to treat LLS in patients who also received upper limb injections (median lower limb dose of 500 U in those treated for ULS and LLS vs 750 U in those treated for LLS alone) may be related to the challenges of trying to remain within the label total body approved dose of 1,500 U. For example, the various health systems of the countries included in this study often require injectors to remain within the recommended ranges for reimbursement purposes (13). However, our data indicate that, even when both upper and lower limbs were injected, the total body dose was often well below 1,500 U, suggesting that other limiting factors (e.g., reimbursement, injector experience, confidence) are at play. The total body dose limitation occurs when patients receive all their injections in 1 session, although current expert opinion is that injectors should prioritize treatment goals at each session, ensuring that an adequate dose is always used to meet that target goal (14, 15). Patients often require repeat treatment cycles, so injectors can plan how to meet different goals over different sessions.

It also is probable that differences in dosing between the 2 subgroups may reflect differences in their clinical presentations. For example, while the dosing in the top 6 muscles was similar between subgroups, the higher doses used in the lower limb only subgroup appear to be driven by the inclusion of less frequently injected muscles, including the hamstrings and the extensor hallucis longus. However, given that striatal toe is a common pattern of LLS (3), the low proportion of extensor hallucis longus injections was surprising. Future analyses of our rich dataset will consider the treatment patterns of patients with post-stroke spasticity, where equinovarus predominates, vs spasticity of other aetiologies, as well as the influence of injector experience, a factor previously reported (16).

BoNT-A injections are just one part of spasticity management as there is consensus that BoNT-A injections should always be complemented by a rehabilitation programme (5, 15). The overall therapy time received (about 2 h with a therapist and ≥ 5 h of self-practice per week) can be considered good for a routine real-world practice study. The therapy interventions used were similar to those reported by patients in an online survey (12), with task practice, passive stretching, and strength training being most popular. However, the use of orthotics and/or splinting was lower than expected for a population that can walk. Approximately one-quarter of patients received other pharmacological interventions for spasticity or spasticity-related pain. However, we did not capture whether the pain medications targeted the lower limb. In the ambulatory population, pain usually is caused by walking on a foot that is unstable when weightbearing, and the pain usually decreases if the foot or toes are straightened. We previously have reported that 70–80% of pain goals were achieved with BoNT-A injections (7).

Strengths of this study lie in its population size and routine real-world practice nature. One limitation was that, while the participating countries were chosen to be broadly reflective of current international practice, important regions such as Asia (where dosing practices may be lower [17]) were not included. Also, studying only ambulatory stroke survivors may have skewed the resulting limb deformities to equinovarus, while other limb deformities may be less well represented. One final limitation, due to the observational nature of the study, is the level of missing data, particularly with regard to therapy interventions.

In summary, this study provides relevant data on how a diversity of muscle patterns are currently treated in routine practice where the primary goal was related to the lower limb. Our findings highlight important issues for further education, debate, and study, such as potential underdosing for optimizing duration of effect, lower limb goals, and the need to balance upper and lower limb priorities when devising a treatment plan.

## Supplementary Material

MUSCLE SELECTION AND DOSING IN PATIENTS UNDERGOING TREATMENT WITH ABOBOTULINUMTOXINA FOR LOWER LIMB SPASTICITY IN REAL-WORLD PRACTICE

## AboLiSh study group

Bozena Adamkiewicz, Arun Aggarwal, Etienne Allart, Claire Aymard, Ziyad Ayyoub, Tobias Bäumer, James Beegan, Francois Boyer, Yuxi Chen, Asare Christian, Regina Chueire, Salvatore Cottone, Edward Dabrowski, Claire Delleci, Ana Paula dos Santos, Steven Edgley, Philippe Gallien, Fatma Gul, Vadim Gusev, Denis Karpov, Ayanna Kersey-McMullen, Dina Khasanova, Svetlana Khatkova, Katja Kollewe, Alexey Korenko, Andrey Korolev, Dariusz Koziorowski, Iwona Jastrzebska Kurkowska, Giacomo Lus, Giorgio Maggioni, Katarzyna Marianska, Vladimir Mikhailov, Cornelia Möbius, Rachael Nunan, Colleen O ʼConnell, Atul Patel, Marcello Riberto, Marcello Romano, Lalith Satkunam, Alexis Schnitzler, Agnieszka Slowik, Nicola Smania, Marsha Smith, Jessica Tate, Martin Todd Taylor, Ip Chi Wang, Jörg Wissel.

## Data sharing

Qualified researchers may request access to patient-level study data that underlie the results reported in this publication. Additional relevant study documents, including the clinical study report, study protocol with any amendments, annotated case report form, statistical analysis plan, and dataset specifications may also be made available. Patient-level data will be anonymized, and study documents will be redacted to protect the privacy of study participants.

Where applicable, data from eligible studies are available 6 months after the primary manuscript describing the results has been accepted for publication. Further details on Ipsen’s sharing criteria, eligible studies, and process for sharing are available here: https://vivli.org/members/ourmembers/. Any requests should be submitted to www.vivli.org for assessment by an independent scientific review board.
