# Supplementary material for: MUSCLE SELECTION AND DOSING IN PATIENTS UNDERGOING TREATMENT WITH ABOBOTULINUMTOXINA FOR LOWER LIMB SPASTICITY IN REAL-WORLD PRACTICE
Source: J Rehabil Med. 2025 Feb 7;57:42605. doi: 10.2340/jrm.v57.42605 (PMC11836465; doi:10.2340/jrm.v57.42605)
Supplement: MUSCLE SELECTION AND DOSING IN PATIENTS UNDERGOING TREATMENT WITH ABOBOTULINUMTOXINA FOR LOWER LIMB SPASTICITY IN REAL-WORLD PRACTICE [file JRM-57-42605-s1.pdf]

Supplementary material has been published as submitted. It has not been copyedited, or typeset by Journal of Rehabilitation Medicine

**Table SI.** Patient baseline characteristics (18)

| Parameter | N=384 |
|---|---|
| Age (years); mean ± SD | 53.9 ± 13.8 |
| Sex; n (%) Male | 255 (66.4%) |
| Diagnosis of condition leading to spasticity; n (%) | |
| Acquired brain injury (stroke/trauma/other) | 374 (97.4%) |
| Spinal cord injury | 8 (2.1%) |
| Other | 2 (0.5%) |
| Aetiology; n (%) | |
| Vascular (infarct or haemorrhage) | 328 (85.4%) |
| Trauma | 44 (11.5%) |
| Hypoxic | 1 (0.3%) |
| Inflammatory | 5 (1.3%) |
| Tumour | 2 (0.5%) |
| Degenerative | 2 (0.5%) |
| Other | 2 (0.5%) |
| Affected by upper limb spasticity; n (%) | |
| Dominant arm | 156 (40.6%) |
| Non-dominant arm | 170 (44.3%) |
| Both | 2 (0.5%) |
| No | 56 (14.6%) |
| Time since onset of the event leading to lower limb spasticity (years) | |
| Mean ± SD | 6.9 ± 7.9 |
| Median [Q1, Q3] | 4.7 [1.7, 8.6] |
| Prior BoNT treatment; n (%) | |
| BoNT naïve | 96 (24.0%) |
| Previously treated | 288 (76.0%) |
| Time interval between onset of event and first BoNT injection (years) | N=309 |
| Mean ± SD | 3.6 ± 6.9 |
| Median [Q1, Q3] | 1.3 [0.6, 3.3] |
| Use of concomitant medications related to spasticity; n (%) | 98 (25.5%) |
